# Supplementary material for: Differential laboratory passaging of SARS-CoV-2 viral stocks impacts the in vitro assessment of neutralizing antibodies
Source: PLoS One. 2024 Jan 25;19(1):e0289198. doi: 10.1371/journal.pone.0289198 (PMC10810540; doi:10.1371/journal.pone.0289198)
Supplement: S1 Fig — (DOCX) [file pone.0289198.s001.docx]

A
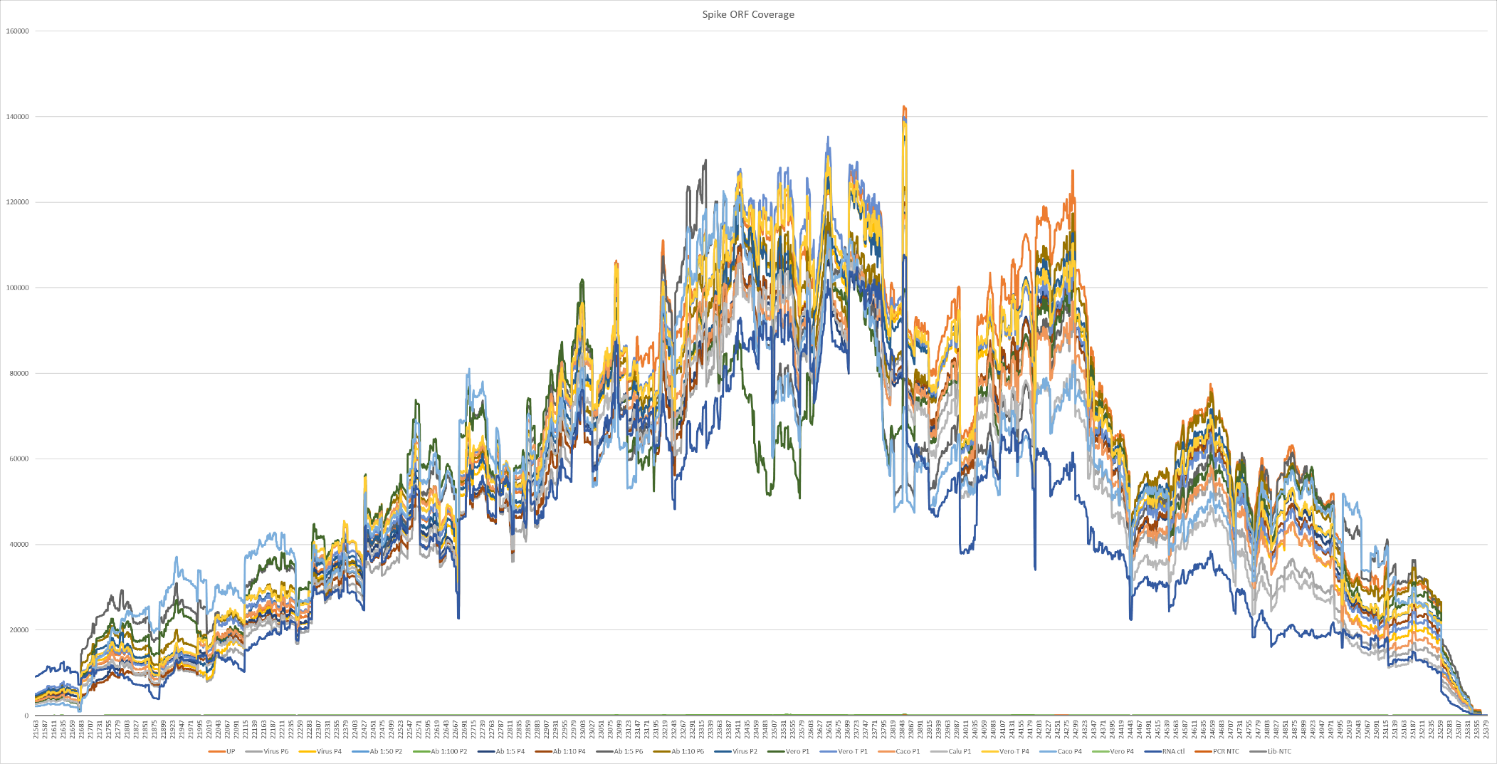


B
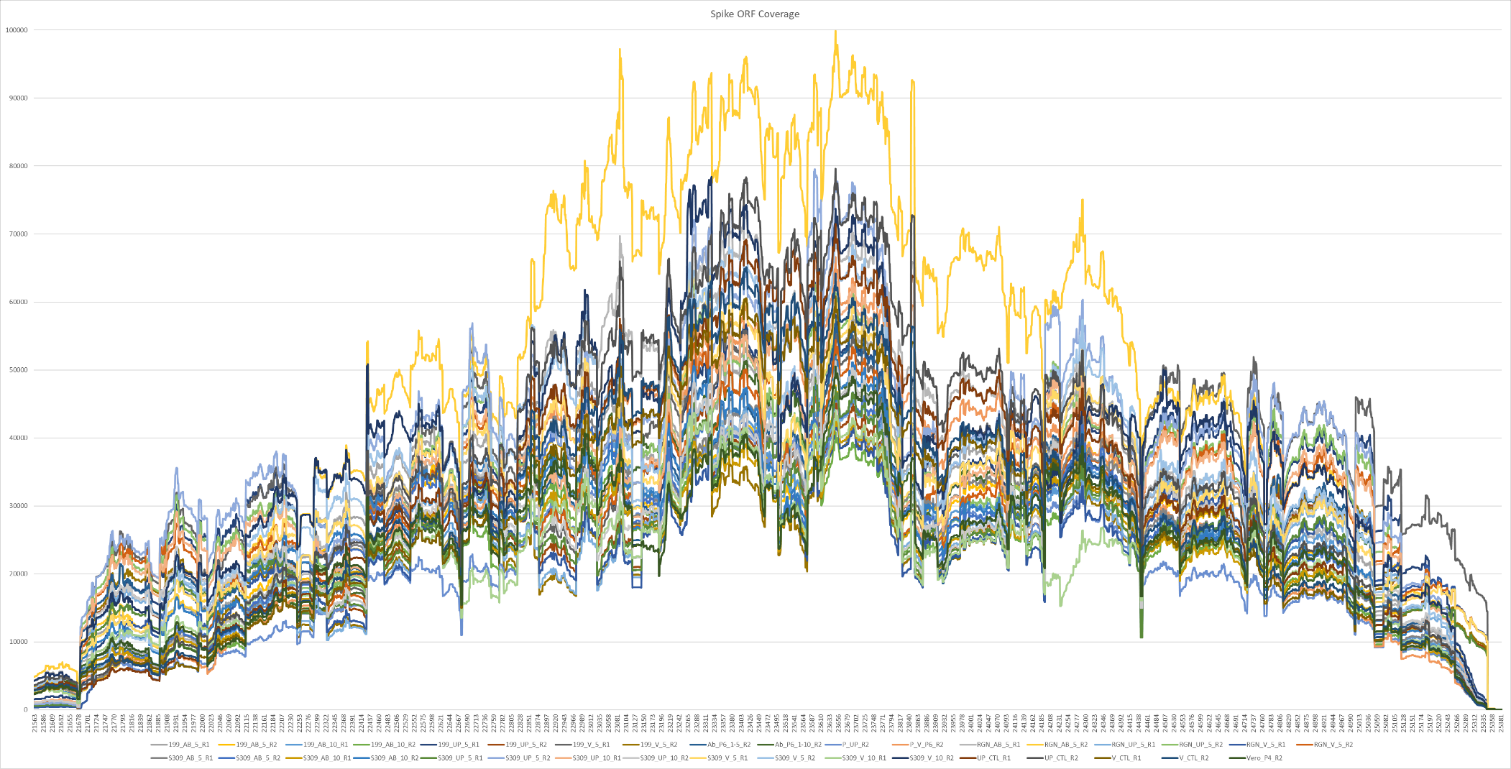


**Figure S1.** **Sequence read coverage depth for sequences derived from neutralization assay**. A) Sequencing Run 1. Note, NTC yielded just 11 mapped reads. B) Sequencing Run 2. No reads mapped for the NTC.
