# Supplementary figures and images for: Differential laboratory passaging of SARS-CoV-2 viral stocks impacts the in vitro assessment of neutralizing antibodies
Source: PLoS One. 2024 Jan 25;19(1):e0289198. doi: 10.1371/journal.pone.0289198 (PMC10810540; doi:10.1371/journal.pone.0289198)

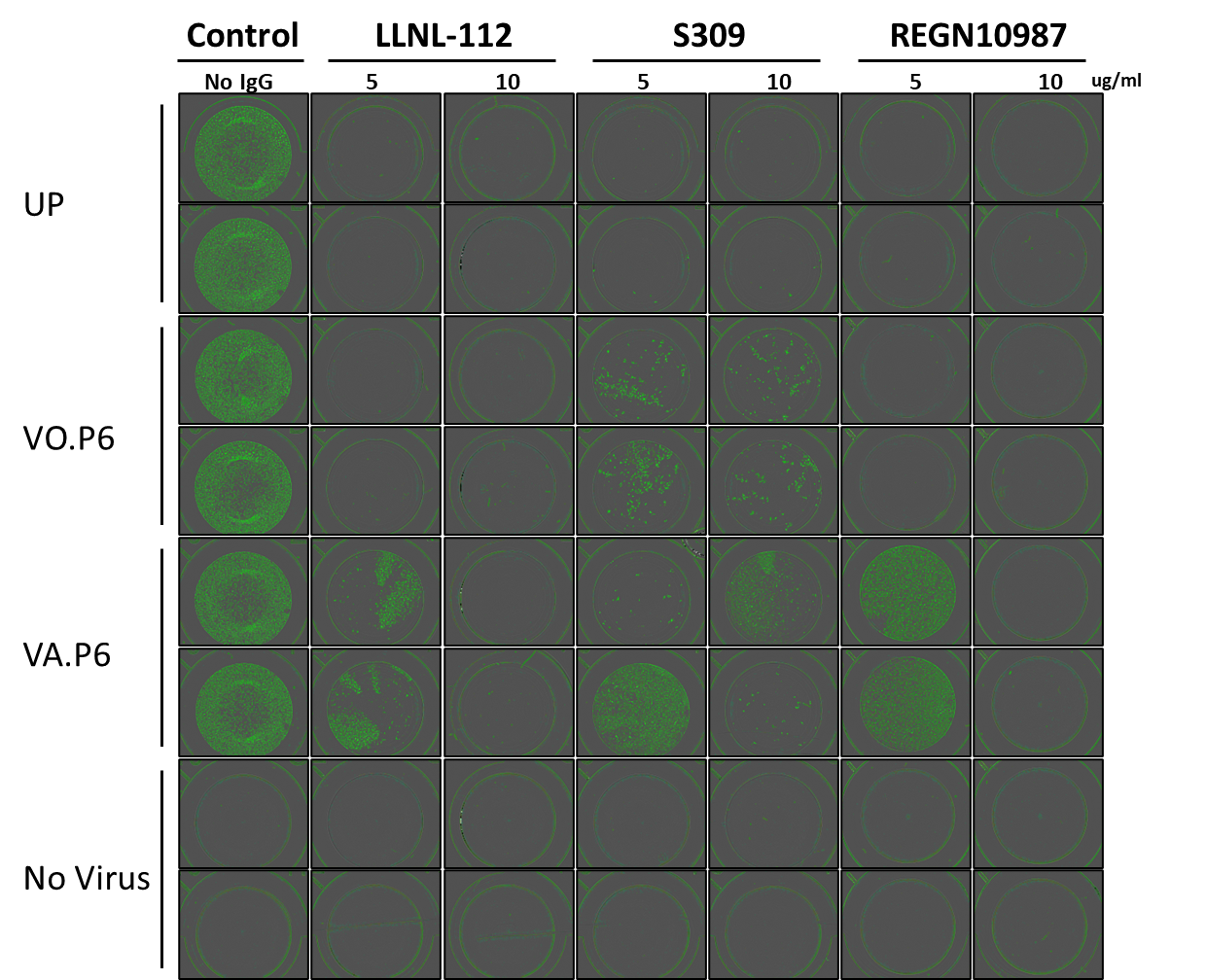


**Figure S2.** **Neutralization assay, GFP expression results**.

Supplement: S2 Fig — (DOCX) [file pone.0289198.s002.docx]

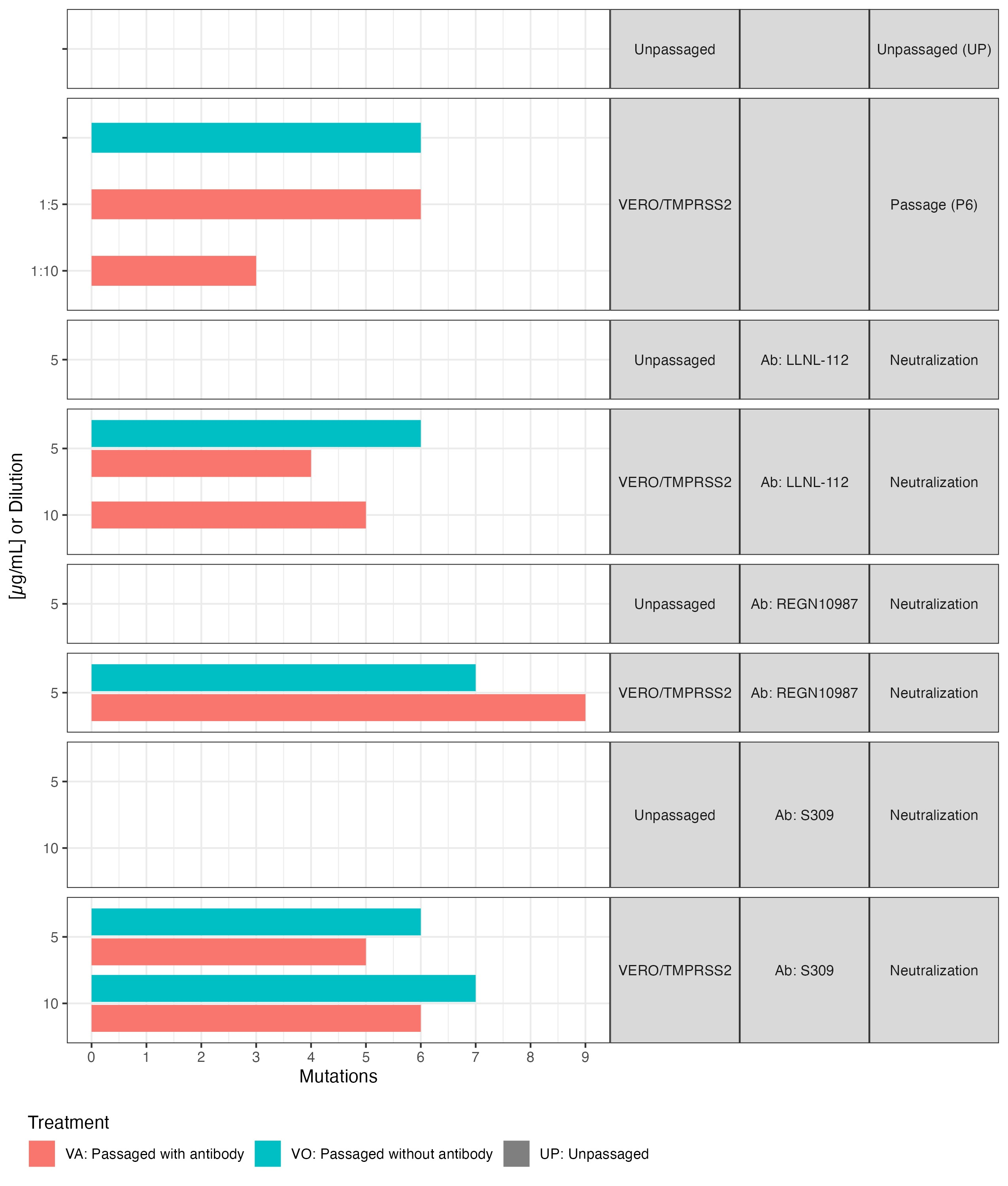


**Figure S4: Number of mutations per sample**

Supplement: S4 Fig — (DOCX) [file pone.0289198.s004.docx]
