# Supplementary material for: Differential laboratory passaging of SARS-CoV-2 viral stocks impacts the in vitro assessment of neutralizing antibodies
Source: PLoS One. 2024 Jan 25;19(1):e0289198. doi: 10.1371/journal.pone.0289198 (PMC10810540; doi:10.1371/journal.pone.0289198)
Supplement: S3 Fig — (DOCX) [file pone.0289198.s003.docx]

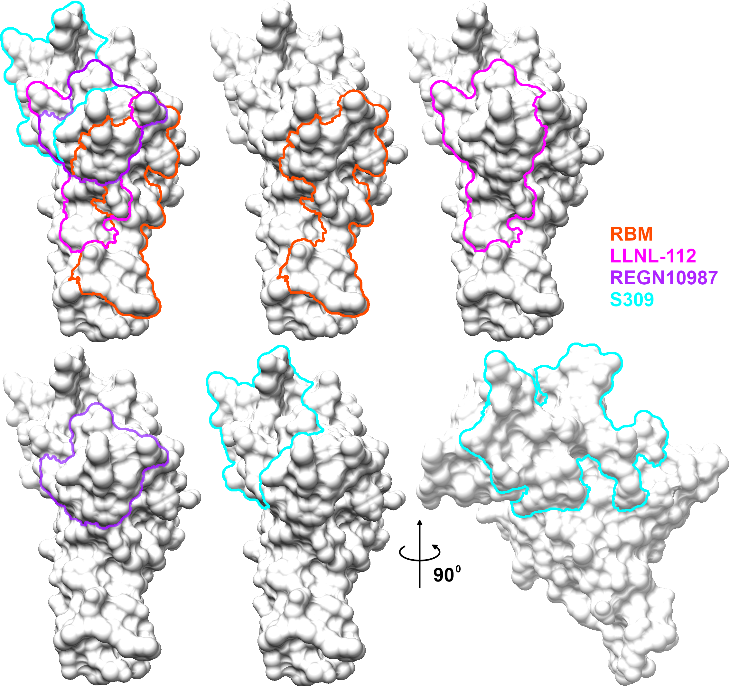


**Figure S3.** **Comparison of epitope regions for three SARS-CoV-2 neutralizing antibodies and the hACE2 receptor binding motif (RBM).** The epitopes are in close proximity (S309) or overlapping (REGN10987 and LLNL-122) the RBM, which likely accounts for the neutralization effects of these antibodies. The epitope regions and the RBM are outlined on the molecular surface of the receptor binding domain (RBD) of SARS-CoV-2 SPIKE protein. The outlined regions enclose all atoms in any residue that has any atom within 5 Å of any atom of the corresponding antibody and contributes to the molecular surface. The RBD is shown in the same orientation for all panels except for the last panel, which is oriented to show a more complete view of the s309 epitope. This view is 90° around the vertical axis with the right side of the molecule rotated away and the left rotated toward the viewer. This figure was generated using Chimera and GIMP.
