# Supplementary material for: Differential laboratory passaging of SARS-CoV-2 viral stocks impacts the in vitro assessment of neutralizing antibodies
Source: PLoS One. 2024 Jan 25;19(1):e0289198. doi: 10.1371/journal.pone.0289198 (PMC10810540; doi:10.1371/journal.pone.0289198)
Supplement: S5 Fig — (DOCX) [file pone.0289198.s005.docx]

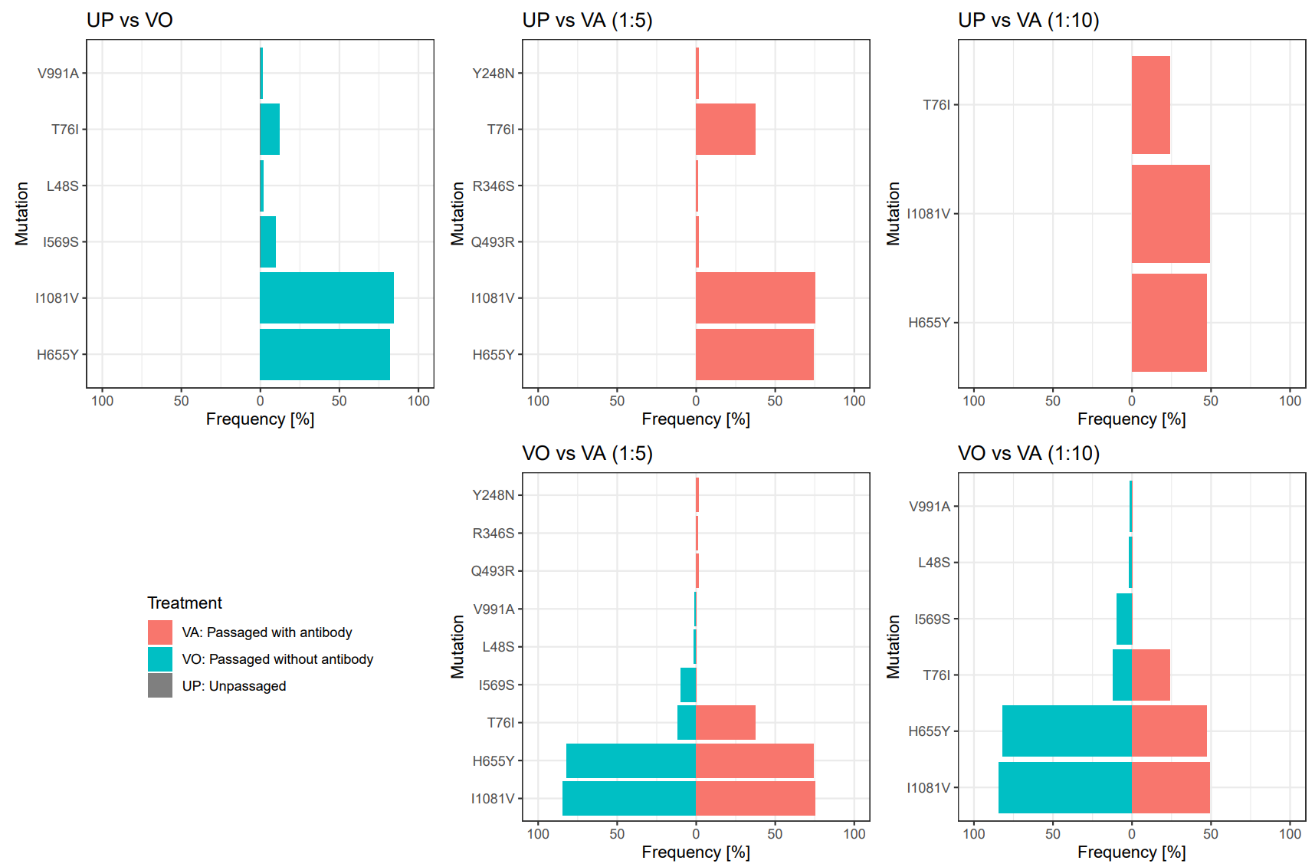


**Figure S5. Frequency of mutations detected in samples passaged in different cell lines and with and without antiserum selection pressure.**
