# Supplementary material for: Differential laboratory passaging of SARS-CoV-2 viral stocks impacts the in vitro assessment of neutralizing antibodies
Source: PLoS One. 2024 Jan 25;19(1):e0289198. doi: 10.1371/journal.pone.0289198 (PMC10810540; doi:10.1371/journal.pone.0289198)
Supplement: Supplementary file 5 [file pone.0289198.s006.docx]

Table S1. Mutation distribution among samples passaged with or without antibody selection pressure. The frequency of detection (%) of the mutations detected at >1% in antisera selection samples and in samples from the neutralization assay are shown for comparison. Data from virus only passaged samples (no antibody selection) are shown in blue cells and data from virus and antiserum passaged samples are in yellow shaded cells. Top row cells shaded grey signify columns with data from both virus only and antibody selection.

	Antiserum or mAb (ng)	V47I	L48S	H66R	G72R	T76I	T167I	L242P	H245Y	S247N	Y248N	E340A	R346S	K378E	L390F	K444N	K444T	G446V	T478K	Q493R	I569S	H655Y	E661G	L945F	L977F	N978K	S982L	D985G	V991A	S1003I	I1081V	R1091H	I1216V
CELL PASSAGE																																	
UP	None																																
VERO P4	None																																
VERO-T P4	None		1.3			11.7																2.5									2.0		
CACO P4	None																																
ANTISERUM PRESSURE																																	
P_VO_P6	None		1.8			12.0															9.7	82.0							1.3		84.4		
VA_P6_1-10	Antiserum			1.4		37.4		1.2		1.6	1.6		1.4							1.7		74.3									75.5		2.7
VA_P6_1-5	Anitserum			10.1		24.0													1.5			47.1									49.3		
NEUT ASSAY																																	
mAB LLNL-112																																	
LLNL-112_UP_5	mAB 199 (5)																																
LLNL-112_V_5	mAB 199 (5)		1.8			12.9															8.8	83.1							1.2		83.2		
LLNL-112_AB_5	mAB 199 (5)					8.6							56.7									88.9									89.1		
LLNL-112_AB_10	mAB 199 (10)					60.9										52.2				58.4		33.8									36.0		
mAb REGN10987																																	
REGN10987_UP_5	REGN10987 (5)																																
REGN10987_V_5	REGN10987 (5)		4.0			11.6															9.7	83.1					1.1		2.0		83.1		
REGN10987_AB_5	REGN10987 (5)					20.0			15.7		17.2					31.2	16.0	40.8		16.0		80.3									82.1		
mAb S309																																	
S309_UP_5	S309 (5)																						5.8	2.5									
S309_UP_10	S309 (10)				3.3																												
S309_V_5	S309 (5)					3.7	1.3							1.8	2.6						46.6	90.0			18.0	5.7		1.1			90.0	2.9	
S309_V_10	S309 (10)	1.3	5.1		1.1	8.3															41.5	89.3					14.0		16.0	1.4	90.0		
S309_AB_5	S309 (5)					10.1						57.1	1.1									87.6									88.7		
S309_AB_10	S309 (10)			1.1		13.6				1.5	1.2	30.0								1.1		83.1									84.0		2.0
